# External facilitators’ perceptions of internal facilitation skills during implementation of collaborative care for mental health teams: a qualitative analysis informed by the i-PARIHS framework

**DOI:** 10.1186/s12913-020-5011-3

**Published:** 2020-03-04

**Authors:** Samantha L. Connolly, Jennifer L. Sullivan, Mona J. Ritchie, Bo Kim, Christopher J. Miller, Mark S. Bauer

**Affiliations:** 1 Center for Healthcare Organization & Implementation Research, VA Boston Healthcare System, 150 S. Huntington Ave., Building 9, Room 208C, Boston, MA 02130 USA; 2 Department of Psychiatry, Harvard Medical School, Boston, MA USA; 3 Department of Health Law, Policy and Management, Boston University School of Public Health, Boston, MA USA; 4 VA Quality Enhancement Research Initiative (QUERI) Program for Team-Based Behavioral Health, Central Arkansas Veterans Healthcare System, North Little Rock, AR USA; 5 Department of Psychiatry, University of Arkansas for Medical Sciences, Little Rock, AR USA

**Keywords:** Internal facilitators, External facilitators, Blended facilitation, i-PARIHS framework

## Abstract

**Background:**

Facilitation is a key strategy that may contribute to successful implementation of healthcare innovations. In blended facilitation, external facilitators (EFs) guide and support internal facilitators (IFs) in directing implementation processes. Developers of the i-PARIHS framework propose that successful facilitation requires project management, team/process, and influencing/negotiating skills. It is unclear what IF skills are most important in real-world settings, which could inform recruitment and training efforts. As prior qualitative studies of IF skills have only interviewed IFs, the perspectives of their EF partners are needed. Furthermore, little is known regarding the distribution of implementation tasks between IFs and EFs, which could impact sustainability once external support is removed. In the context of an implementation trial, we therefore: 1) evaluated IFs’ use of i-PARIHS facilitation skills, from EFs’ perspectives; 2) identified attributes of IFs not encompassed within the i-PARIHS skills; and 3) investigated the relative contributions of IFs and EFs during facilitation.

**Methods:**

Analyses were conducted within a hybrid type II trial utilizing blended facilitation to implement the collaborative chronic care model within mental health teams of nine VA medical centers. Each site committed one team and an IF to weekly process design meetings and additional implementation activities over 12 months. Three EFs worked with three sites each. Following study completion, the EFs completed semi-structured qualitative interviews reflecting on the facilitation process, informed by the i-PARIHS facilitation skill areas. Interviews were analyzed via directed content analysis.

**Results:**

EFs emphasized the importance of IFs having strong project management, team/process, and influencing/negotiating skills. Prior experience in these areas and a mental health background were also benefits. Personal characteristics (e.g., flexible, assertive) were described as critical, particularly when faced with conflict. EFs discussed the importance of clear delineation of EF/IF roles, and the need to shift facilitation responsibilities to IFs.

**Conclusions:**

Key IF skills, according to EFs, are aligned with i-PARIHS recommendations, but IFs’ personal characteristics were also emphasized as important factors. Findings highlight traits to consider when selecting IFs and potential training areas (e.g., conflict management). EFs and IFs must determine an appropriate distribution of facilitation tasks to ensure long-term sustainability of practices.

**Trial registration:**

Clinicaltrials.gov, September 7, 2015, #NCT02543840.

## Background

There is a crucial need within the field of implementation science to better understand the specific mechanisms that contribute to successful implementation of healthcare innovations. Gaining an understanding of which implementation strategies do and do not work, and at what “dose,” will ideally strengthen study designs, increase uptake of innovations, and ultimately improve health outcomes [1, 2]. One key strategy needing examination is facilitation, the process of aiding uptake of innovations by helping individuals and teams to understand change processes and achieve implementation goals [3].

The importance of facilitation is outlined in the integrated Promoting Action on Research Implementation in Health Services (i-PARIHS) framework. The i-PARIHS framework proposes that successful implementation is the result of facilitation of the innovation among its identified recipients, occurring within the local, organizational, and external context [4]. I-PARIHS therefore emphasizes four core constructs: qualities of the innovation itself, characteristics of the recipients of the given innovation, contextual factors at the micro, meso, and macro levels, and the process of facilitation. Importantly, facilitation is referred to as the “active ingredient” within the i-PARIHS framework, in that it is ultimately responsible for providing implementation strategies and actions that are tailored to the innovation, recipients, and context [4]. The current study focuses on this construct of facilitation, given its central role in influencing implementation outcomes.

Facilitation can take various forms. External facilitators (EFs) are individuals from outside of the implementation setting, often with specialized training in implementation facilitation, who serve an outreach role, providing guidance and support to individuals and/or teams tasked with adopting a given innovation. Within a blended facilitation model, EFs work in collaboration with internal facilitators (IFs)—individuals from within the local context—to help direct the implementation process and foster the development of facilitation skills within IFs [3, 5–8]. EFs and IFs are therefore critical in having a nuanced understanding of the factors influencing implementation of an innovation in a given context, and what processes need to change in order to increase uptake. While previous work has outlined components of facilitation [3, 6], there is a lack of empirical research examining how facilitation skills are actually utilized in real-world settings. This is important to examine among IFs, as it is unclear what skills are deemed most important, which could inform recruitment and training processes. Indeed, IFs may have a major impact on implementation outcomes, including the sustainability of practices once external support is removed.

Therefore, there is a need for more focused qualitative work examining IF characteristics. Previous qualitative studies have examined IFs’ perspectives on their role in the facilitation process, including managing projects, communicating effectively, troubleshooting problems, and supporting their team [9–11]. These studies employed rigorous designs involving triangulation between multiple sources of data including individual interviews and focus groups with IFs and activity logs. However, having IFs evaluate their own performance may in part limit the scope of study findings, given that individuals may be less likely to acknowledge or be aware of potential strengths or areas for improvement. Therefore, it is necessary to examine IF characteristics from the additional perspective of their EF partners, who worked closely with IFs during implementation. In addition, little is known regarding EF/IF dynamics during the facilitation process. For instance, the degree to which EFs guide and support IFs in the completion of facilitation tasks, versus EFs completing project-related tasks themselves, varies considerably across models of facilitation and real-world applications [3, 6, 8, 12]. This interplay may also have major effects on the long-term sustainability of implementation practices.

We sought to examine these questions within the context of the i-PARIHS framework. According to i-PARIHS developers, facilitators must demonstrate skills in several areas, including: project management and improvement (e.g., helping sites adhere to project deadlines); team and process (e.g., helping teams to problem-solve or come to consensus), and influencing and negotiating (e.g., advocating for the team when meeting with mental health leadership [4]). Indeed, these three skill areas were among the facilitator attributes discussed in reviews of the theory- and research-based facilitation literature [13, 14]. Although perhaps emphasized less often than task-related skills [15], personal characteristics such as being motivated, flexible, and assertive have also been identified as important facets of facilitation [3, 13].

Thus, there is an opportunity to better understand IF skills through the lens of i-PARIHS and from the unique perspective of their EF partners. The current qualitative study, undertaken in the context of a hybrid type II implementation trial using a blended facilitation strategy within outpatient mental health teams, aimed to: 1) examine IFs’ use of i-PARIHS facilitation skills, from EFs’ perspectives; 2) identify additional attributes of IFs not encompassed within i-PARIHS skills; and 3) investigate the relative contributions of IFs and EFs during implementation, to better understand sustainability of implementation processes. The current research examines IF skills from the important and understudied perspective of their EF partners, and to our knowledge is the first to examine real-world IF characteristics as they relate to i-PARIHS facilitation recommendations, representing significant contributions to the literature.

## Methods

### Study setting

The current study uses data collected from a randomized stepped wedge hybrid type II implementation trial conducted across nine medical centers within the US Department of Veterans Affairs (VA) (see Bauer et al., 2015 [16] for a full description of study design and methods and Bauer et al., 2019 [17] for implementation outcomes). Each medical center identified an interdisciplinary treatment team within their general mental health clinic that would undergo implementation of the Collaborative Chronic Care Model (CCM) to improve care processes. The CCM is an evidence-based approach to structuring care for chronic conditions including mental health disorders [18, 19]. It includes domains such as increasing patients’ self-management skills, improving team-based communication among providers, and securing the support of mental health leadership in these efforts. Medical centers were recruited through national VA publicity. Sites committed their treatment team to weekly hour-long process design meetings over the 12-month project period, in addition to identifying a staff member willing to serve as an IF for the duration of the project at 10% effort, funded by the local facility. IFs did not need to have prior experience within the mental health team they were assigned to, nor were they required to have a mental health background. Three EFs were self-selected from within the study team receiving grant funding to conduct this trial (BK, CJM, MSB); each had expertise in the CCM and had completed a structured intensive facilitation training [20]. Each EF partnered with three of the included medical centers. This study was a combined program evaluation and research project; it was reviewed by the VA Central Institutional Review Board (IRB), and implementation efforts and implementation-related measures were determined to be exempt from IRB review.

### Facilitation processes

The study used a blended internal-external facilitation model informed by the Replicating Effective Programs (REP) framework stages of implementation including assessing pre-conditions, pre-implementation preparation, implementation, and maintenance [5, 21]. At each site, EFs completed a pre-site visit assessment; a 1.5-day kickoff site visit; 6 months of weekly videoconferences or phone calls with the treatment team and IF; weekly individual meetings and ad hoc communications with the IF; and 6 months of step-down facilitation activities on an as-needed basis. EFs guided the implementation process with a structured workbook aligned with the elements of the CCM, allowing IFs to engage in assessment and undertake process redesign based on goals identified within their team (e.g., to increase patient involvement during treatment planning; to improve communication with other clinics).

### Data collection

In 2018, following the end of the implementation trial, the three EFs completed semi-structured interviews assessing their impressions of IF skills and EF/IF dynamics during the project. The interview guide was informed by i-PARIHS facilitation recommendations and included the following areas: project management and improvement; team and process; and influencing and negotiating skills [see Supplementary File]. Personal characteristics were also included, given literature discussing their key role within facilitation [3, 13, 15]. An expert in facilitation (MJR) reviewed these skill areas and provided critical feedback during interview construction. The first author (SLC), who is a research colleague of the three EFs, conducted all interviews. EFs were asked to comment on important IF skills within each area. They were first asked to comment on these areas on a broad level, and then were prompted to elaborate on any specific differences they observed among the IFs they worked with. EFs then provided any additional feedback not encompassed within the given skill areas. Interviews were audio recorded and professionally transcribed verbatim.

### Data analysis

Transcripts were analyzed in NVivo 10 via directed content analysis [22, 23] informed by the facilitation skills identified by i-PARIHS developers, utilizing a priori codes representing the five areas outlined above: project management and improvement skills; team and process skills; influencing and negotiating skills; personal characteristics; and the role of EFs in relation to IFs. The first and second authors read all transcripts and assigned codes to the data, allowing for the emergence of additional codes. The authors then met to compare their code assignments, discuss any coding discrepancies, and achieve consensus for each transcript.

We took several steps to strengthen the credibility of analyses, in accordance with the Standards for Reporting Qualitative Research; SRQR [24]. The first and second author, who conducted the data analysis, have substantial experience within the fields of mental health and implementation science research within VA. This prolonged engagement allowed for improved data interpretation abilities and may have increased EFs’ comfort and willingness to disclose information during the semi-structured interview. All research procedures and analytic decisions were thoroughly documented and discussed during the development of this work. The first and second author met to compare codes and achieve consensus in an effort to reduce bias. The interpretation of findings was also reviewed by the third author, an expert in facilitation who did not participate in the research project, to further reduce bias. Member checking was also employed, such that the description of facilitation activities and interpretation of results was reviewed by the three EFs. The EFs, who are co-authors on this work, read all drafts of the manuscript and provided feedback during a series of in-person meetings, which greatly informed subsequent edits. All EFs read the final submitted draft and approved of its characterization of their perspectives.

## Findings

The three EFs were health services researchers (health systems engineer, clinical psychologist, psychiatrist). Two sites transitioned between IFs during the implementation year and therefore had two IFs, resulting in 11 IFs having participated in the project across nine sites. Five IFs (45%) were social workers, four (36%) were clinical psychologists, one (9%) was an advanced practice registered nurse, and one (9%) was an administrator; eight (73%) had formal supervisory or leadership roles within mental health. The nine participating sites were distributed across the northeastern, midwestern, and southern United States. We present findings within the five assessed areas below: project management and improvement skills; team and process skills; influencing and negotiating skills; personal characteristics; and EF/IF dynamics. Table 1 summarizes main findings.

**Table 1:** Summary of key findings

Facilitation area	Key factors
IF project management and improvement skills	- Clear goal-setting
	- Step-by-step planning and delegation of tasks
	- Organization and attention to detail
	- Clearly defined roles
	- Willing to complete frontline work
	- Adequate bandwidth to devote to project
	- Frequent follow-up and tracking of progress
	- Prior project management experience
IF team and process skills	- Thought leader, champion, model, guide, motivator
	- Instills sense of teamwork and unity
	- Respected and trusted by team, has sense of authority
	- Well-established in team prior to implementation project
	- Formal supervisory or leadership role
	- Clear and transparent communication
	- Seeks team’s input and feedback
	- Effective management of team tensions and conflict
IF influencing and negotiating skills	- Higher level leadership position, or connections to these levels
	- Prior establishment within mental health
	- Successful advocate for team, ability to secure leadership buy-in
	- Understanding of contextual factors (e.g., relation to other service lines)
	- Willing to address conflicts with service line or leadership
	- Prior influencing and negotiating experience
IF personal characteristics	- Warm, personable, outgoing, optimistic, self-motivated
	- Practical, goal-oriented, patient, non-punitive
	- Confident, assertive
	- Natural leader and problem-solver
	- Flexible, open, willing to take a “leap of faith” and trust a new process
	- High impetus for change
	- Willing to ask for help and acknowledge weaknesses
	- Willing to approach conflict, respond to challenging feedback from team members
EF/IF dynamics	- EF serves as expert, consultant, and educator regarding intervention content and implementation process
	- IF serves as expert on local needs, policy, and culture
	- Goal of tapering EF effort over time as IFs gain experience
	- EFs make substantial contribution to project deliverables, frequent follow-up with IFs
	- Lack of IF/EF role clarity; EF could have been more direct in working with IFs to shift balance of responsibilities more towards IF as implementation progressed

### Project management and improvement skills

The EFs all described the need for IFs to set clear goals when working to implement the CCM within their mental health team. One noted the importance of this process on overall team buy-in: *“Yes, it might have taken a little bit of extra time and effort on [the IF’s] part…[helping] people not lose sight of what it is that they are spending at least one hour a week on doing in terms of process redesign. [EF 1]”* Having clearly defined roles within the team, including an understanding of the responsibilities of the IF and EF was described as important, particularly as this related to being able to delegate tasks and problem-solve effectively. One EF reflected on the style of an effective IF:

*[The IF] was definitely taking care of many things for the service, but [they] asked the team for a lot of help…I think that kind of problem-solving, but not overly solving by one’s self [is important] …being able to identify problems before jumping to conclusions as to how they should be solved. [EF 1].*

Tracking progress over time and ensuring continuity of project goals was described as crucial: *“the ability to remember week two what you promised to do in week one... It was harder when people let fall off the radar screen what they had planned to do. [EF 2]”* EFs emphasized that, given the busy schedules of the clinicians on each team, IFs could not expect that decisions made at a one-hour meeting would be remembered or acted upon without active follow-up and engagement on the IFs’ part; *“[the IF] had to have a mindset that [they] weren’t above doing the frontline integrated work. [EF 2]”* The EFs described their IFs as having prior experience in areas such as process redesign and improvement, and viewed this as a strength.

One EF emphasized the need for IFs to provide adequate guidance to team members, particularly during earlier stages of implementation, versus allowing for too much autonomy too soon:

*It’s true that once the team is at a point where they are grasping what it means to self-drive improvement and continuously think about different ways to innovatively do things for the team, they are really ready to run as their own machine…but before they get there, slightly more direct ways of managing [would be helpful]. [EF 1].*

The EFs noted that IFs typically juggled multiple responsibilities, including one case in which an IF was detailed to another initiative, which impacted the availability of IF time for facilitation activities. All EFs described the importance of IFs maintaining frequent and timely communication with EFs, while noting that other job duties and conflict within the team could make it difficult to sustain this type of regular correspondence.

### Team and process skills

IFs were described as thought leaders, champions, models, and guides who introduced the team to new processes step by step in a collaborative fashion. Two EFs described the importance of the IF instilling a sense of teamwork and unity; one EF emphasized that this was particularly important during periods of uncertainty within their site, such that the team felt that they *“still have each other…that there are some gains to be made even though [certain] external things can’t change. [EF 2]”* Being respected and trusted by team members was highlighted as a key strength, as one EF noted:

*I think what made [that IF] most successful was the fact that [the team] really saw the internal facilitator as somebody who was working in their best interest, being able to trust the internal facilitator when they said, “Trust me about this, I’m trying to look out for you.” [EF 1].*

The EFs agreed on the importance of the IF serving a motivating role, which one EF described as:

*Being able to encourage participation, encourage attendance in a way that feels non-punitive but still gets the job done…and keeping that participation and engagement going outside of the team meeting...being able to drum up excitement even though people are in the midst of a stressful work environment. [EF 3].*

The EFs agreed that it was beneficial if the IF was well-established within their clinic prior to project implementation, including having a formal supervisory or leadership role, as team members were already accustomed to turning to the IF for guidance. However, all EFs discussed circumstances in which IFs were able to gain the respect of their team despite being from a different discipline or from outside of the mental health service. They each cited instances in which having the support of a well-regarded, established member of the team benefited IFs entering from differing services or disciplines.

All EFs described team-based conflicts which at times affected meeting dynamics or team productivity. EFs emphasized the importance of IFs communicating clearly and openly with team members to foster positivity and avoid potential misunderstandings. EFs also noted some circumstances in which IFs struggled to gain support from team members during the project, including those from disciplines different from their own. For instance, if an IF had difficulty gaining buy-in from the psychiatry lead, it could be more challenging to encourage participation from other team psychiatrists.

### Influencing and negotiating skills

EFs agreed that being positioned at a higher level of mental health leadership, or having connections to these higher levels or to other clinics, was a benefit for IFs by helping to improve communication channels and earn respect from the team. Having prior experience negotiating across levels and between clinics was also viewed positively. EFs remarked on the skill with which some IFs advocated for their teams. One described an experienced IF who navigated complex cross-clinic dynamics to communicate their team’s needs and concerns to leadership. Another EF described how an effective IF shared their team’s progress to further gain support and buy-in:

*The internal facilitator not only passed information up the chain but also did what they could to engage leadership, so that the team felt like leadership was paying attention to them in a positive way. For example, inviting leadership in to hear a report from the team on changes that they made. That was seen as a positive step. [EF 3].*

One EF described several instances in which IFs effectively advocated for their team when experiencing conflict with other clinics or higher levels of leadership. EFs agreed that having a strong grasp of context was important, such as understanding service line dynamics, as well as identifying and valuing key stakeholders across clinics and disciplines who ultimately affect team functioning. One EF noted the importance of IFs knowing what team processes were and were not within the team’s control, to help increase the likelihood of success during process improvement efforts:

*Revamping the intake process…is often decided at the clinic level. Changing the extent to which providers have control over their scheduling, is not usually decided at the team level. Those are things that are clearly very important to the teams…but if they put a lot of time and effort into considering those issues and then were told no… that kind of hurt. [EF 3].*

IF difficulties securing leadership buy-in were also noted at times. EFs differed in the degree to which they felt this was in part due to reticence on the part of IFs, versus being attributable to larger environmental factors out of the IFs’ control. For instance, one EF discussed the inherent difficulties of influencing and negotiating when an IF was from outside of the mental health service:

*…they typically don’t have a whole lot of leverage or clout with mental health leadership who may ultimately be the ones deciding about resource allocation, whether to assign a provider to the team, whether [a scheduler] can take some time out to attend the team meetings…I would think that it would be easier…if they already had connections to the people who make those decisions. [EF 3].*

### Personal characteristics

EFs described the importance of IF personal characteristics including being warm, personable, outgoing, and natural leaders. Optimism and self-motivation were also seen as benefits, such that IFs did not dwell on failures and persevered despite encountering obstacles. Additional positive traits included being practical, goal-oriented, patient, non-punitive, clear in delegating tasks, and a strong problem-solver. One EF emphasized the importance of the IF being flexible, open to new ideas, and willing to take a *“leap of faith [EF 1]”* in trusting the process and thinking beyond current practices. Another EF described an IF with a clear impetus for change, reflected in the IF’s motivation to accomplish goals in a timely fashion; *“a kind of a willingness to try to make the best of what you have as opposed to waiting for something to change. [EF 2]”* EFs emphasized the need for IFs to be assertive, willing to approach team conflict, and open to potentially challenging feedback from team members.

### EF/IF dynamics

EFs described serving as an expert, consultant, model, and educator, tasked with providing concrete advice and direction to IFs. This could range from helping to plan and structure team meetings, to assisting in navigating conflict at the team, service line, or leadership level. The degree of on-the-ground guidance also varied across the scope of the project, with EFs describing tapering their effort and presence within team meetings as the IF and their team had made sufficient progress and no longer needed this level of support. All EFs acknowledged that they contributed substantially to producing deliverables, and were frequently following up with IFs to receive updates on progress or to ensure that tasks were completed. They all discussed that there was a need for role clarity between IFs and EFs, and emphasized the importance of IFs taking initiative in completing project tasks, especially in the later stages of facilitation. One EF discussed how the study design influenced expectations regarding the relative effort of IFs and EFs:

*We, as external facilitators, wanted the internal facilitator to be driving the process. But in reality, we are the ones coming into their medical center with extra funding, helping them out. I think it was hard to not view this as an externally-driven process. [EF 3].*

This EF went on to raise concerns regarding the sustainability of practices after external support was removed. EFs acknowledged that they could have been more direct in working with IFs to shift greater responsibility into IFs’ hands during facilitation, which may have helped to direct control away from external supports and over to the local site.

## Discussion

The current study sought to better understand facilitation skills employed during a hybrid type II implementation trial aiming to integrate the CCM into outpatient mental health teams. EFs described the importance of IFs utilizing skills consistent with i-PARIHS facilitation recommendations. With regards to project management and improvement, EFs highlighted the need for IFs to set appropriate goals, clearly define team member roles, delegate tasks effectively, and engage in frequent follow-up. Regarding team and process skills, EFs emphasized the role of IFs as thought leaders and guides who were respected and trusted by their team. Having a sense of authority as well as the ability to communicate clearly and openly with team members were also benefits. Within the area of influencing and negotiating, important IF skills included having strong connections with higher levels of leadership, being advocates for their team, and understanding the context in which implementation was occurring. Having prior experience within these areas, as well as a mental health background, were described as benefits. In addition to the i-PARIHS facilitation skill areas, EFs emphasized the key role of personal characteristics. These traits included being optimistic, flexible, assertive, motivated for change, and willing to address conflicts in a direct and constructive manner.

EFs noted that in addition to IFs’ skills, it is important to consider the role of environmental factors during facilitation. EFs described how processes occurred more smoothly within teams that were cohesive and well-established prior to implementation efforts. As such, it was observed that there was an interactive effect between a given IF and the team that they entered into, and that certain IF styles and personal characteristics would likely fare better or worse within different teams. Similarly, factors such as IFs’ prior experience, or lack thereof, within a given team or service line, were also important to consider when examining facilitation processes.

In general, findings support the importance of i-PARIHS facilitation skills, and therefore are aligned with reviews of the theory and research-based facilitation literature that highlight these key areas [13, 14]. However, as has been noted elsewhere [3, 13, 14], personal characteristics were also seen as critical, and therefore may warrant further emphasis in conceptualizations of internal facilitation. Indeed, it has been argued that IFs’ emotional intelligence may be a central, and often underemphasized, component of their performance within this role [15]. These skills were seen to be especially important in the current study as they related to conflict management, and suggest that specific training and guidance within this domain may be indicated. For instance, the VA-developed Implementation Facilitation Training Manual includes modules specific to responding to resistance to change among key stakeholders, including the use of motivational interviewing techniques [25]. It will be important for future work to continue studying the role of IF personal characteristics, and to consider adapting implementation frameworks to include this critical component of facilitation. Findings emphasize additional areas that may also be important to include in training, such as the role that securing support from leaders of different mental health disciplines can have in increasing buy-in among team members within those disciplines.

With regards to the dynamic between EFs and IFs, EFs noted playing a significant role in managing facilitation tasks. Some EFs noted that they could have been more direct in collaborating with IFs to define their respective roles and set clearer expectations regarding the distribution of responsibilities, particularly as facilitation progressed. EFs noted that this dynamic may affect the long-term sustainability of practices once external research support was removed. Descriptions of facilitation in the literature differentiate between facilitators “doing for others” versus “enabling others,” and note that the degree to which facilitators fall at either end of this spectrum can vary significantly across projects [3, 12]. For example, the VA Implementation Facilitation Training Manual describes EFs as primarily serving a consulting and guiding role, with IFs managing internal implementation tasks over time [25], and a qualitative study of EFs reported that they only occasionally took “direct action” in completing facilitation tasks [8]. Current findings reveal the potential difficulty of shifting facilitation responsibility to IFs, particularly within the context of an externally-funded study in which IFs may expect their research partners to take a more prominent role. These findings emphasize the importance of EFs and IFs clearly defining their respective roles and expectations to ensure the successful transition of facilitation responsibilities. It will be important for future research to examine the effect that varying EF/IF dynamics have on the long-term sustainability of implementation processes.

The current research examines IF characteristics from the understudied perspective of their EF partners, which allows for a unique view of IF roles as compared to that obtained via IF self-report data. Furthermore, it is the first study to examine real-world IF skills within the context of the i-PARIHS facilitation recommendations, in addition to identifying additional factors not encompassed within this framework. Our use of directed content analysis is a strength, in that our analyses were informed and structured by an established implementation framework and the pre-existing IF literature. This directed content approach differs from alternative qualitative methods such as thematic analysis, a more flexible approach which involves identifying themes and patterns in the data and does not require the use of a corresponding model or framework [26]. While thematic analysis has many of its own strengths, our decision to employ a directed content approach allows our findings to contribute to the growing i-PARIHS literature within the field of implementation science.

However, there are several limitations to note. The current design involved interviewing EFs at the end of facilitation. Ideally, data would have been collected throughout this process, including via field notes and periodic interviews, to track changes in perspectives over time and allow for triangulation between multiple data sources. Three EFs were interviewed; although this represents all of the EFs involved in this trial, it is unclear to what extent their viewpoints and experiences are generalizable to other implementation efforts. The EFs were PhD and MD-level health services researchers who received funding from the larger study within which these analyses took place; therefore, their perceptions of IF skills will inevitably have been influenced by their degree of authority and experiences as researchers. Due to limitations in the scope of the study and the extent of commitment pledged by participating sites, we were unable to also interview the IFs, which would have helped to provide balance alongside the EFs’ viewpoints. However, given that multiple prior studies have opted to interview IFs [9–11], the current research aimed to expand upon this work and examine IF characteristics from the novel perspective of their EF partners. Nonetheless, we acknowledge the importance of gaining multiple perspectives when exploring facilitation processes, and agree that future work should aim to include both EF and IF perspectives if possible. Additional, more nuanced analyses could have strengthened current findings and will be important to explore in future work; for instance, further study of the interplay between IFs’ personal characteristics and the unique contexts in which they are situated, as well as how characteristics of the innovation—in this case, the CCM—impact the skills most needed in an IF.

## Conclusions

In sum, key IF skills, according to EFs, are aligned with i-PARIHS facilitation recommendations, but personal characteristics were also seen as critical. When selecting IFs, it is important to consider their skills and prior experience within the areas of project management and improvement, team and process, and influencing and negotiating, in addition to evaluating personal characteristics associated with strong leadership abilities, particularly during conflict. IFs may also benefit from conflict management training to best respond to challenges encountered during facilitation. Furthermore, EFs and IFs should set clear expectations regarding their respective roles throughout the implementation process to ensure long-term sustainability of practices. Future work should continue to draw from established implementation frameworks such as i-PARIHS to evaluate real-world facilitation practices, and to examine their effects on both short and long-term implementation outcomes.

## Supplementary information

**Additional file 1.** Interview Guide.

## Data Availability

A de-identified version of the dataset used during the current study is available from the corresponding author on reasonable request.

## Abbreviations

CCM: Collaborative chronic care model
EF: External facilitator
IF: Internal facilitator
i-PARIHS: Integrated Promoting Action on Research Implementation in Health Services
IRB: Institutional review board
REP: Replicating Effective Programs framework
SRQR: Standards for Reporting Qualitative Research
VA: US Department of Veterans Affairs

## References

[1] Butow P, Shaw J, Shepherd HL, Price M, Masya L, Kelly B (2018). Comparison of implementation strategies to influence adherence to the clinical pathway for screening, assessment and management of anxiety and depression in adult cancer patients (ADAPT CP): study protocol of a cluster randomised controlled trial. BMC Cancer.

[2] Proctor EK, Powell BJ, McMillen JC (2013). Implementation strategies: recommendations for specifying and reporting. Implement Sci.

[3] Harvey G, Loftus-Hills A, Rycroft-Malone J, Titchen A, Kitson A, McCormack B (2002). Getting evidence into practice: the role and function of facilitation. J Adv Nurs.

[4] Harvey G, Kitson A (2015). PARIHS revisited: from heuristic to integrated framework for the successful implementation of knowledge into practice. Implement Sci.

[5] Kirchner JE, Ritchie MJ, Pitcock JA, Parker LE, Curran GM, Fortney JC (2014). Outcomes of a partnered facilitation strategy to implement primary care–mental health. J Gen Intern Med.

[6] Ritchie M, Kirchner J (2017). Facilitation: a key strategy in the field of implementation science. FORUM: VA Office of Research & Development, Health Services Research & Development Service, Center for Information Dissemination and Education Resources.

[7] Ritchie MJ, Parker LE, Edlund CN, Kirchner JE (2017). Using implementation facilitation to foster clinical practice quality and adherence to evidence in challenged settings: a qualitative study. BMC Health Serv Res.

[8] Stetler CB, Legro MW, Rycroft-Malone J, Bowman C, Curran G, Guihan M (2006). Role of “external facilitation” in implementation of research findings: a qualitative evaluation of facilitation experiences in the veterans health administration. Implement Sci.

[9] Dogherty EJ, Harrison MB, Baker C, Graham ID (2012). Following a natural experiment of guideline adaptation and early implementation: a mixed-methods study of facilitation. Implement Sci.

[10] Dogherty EJ, Harrison MB, Graham ID, Vandyk AD, Keeping-Burke L (2013). Turning knowledge into action at the point-of-care: the collective experience of nurses facilitating the implementation of evidence-based practice. Worldviews Evid-Based Nurs.

[11] Kotecha J, Han H, Green M, Russell G, Martin MI, Birtwhistle R (2015). The role of the practice facilitators in Ontario primary healthcare quality improvement. BMC Fam Pract.

[12] Loftus-Hill A, Harvey G (2000). A review of the role of facilitators in changing professional health care practice. RCN Institute.

[13] Dogherty EJ, Harrison MB, Graham ID (2010). Facilitation as a role and process in achieving evidence-based practice in nursing: a focused review of concept and meaning. Worldviews Evid-Based Nurs.

[14] Ritchie MJ (2016). Transferring implementation facilitation knowledge and skills to improve healthcare delivery systems (dissertation).

[15] Elledge C, Avworo A, Cochetti J, Carvalho C, Grota PJC (2018). Characteristics of facilitators in knowledge translation: an integrative review. Collegian.

[16] Bauer MS, Miller C, Kim B, Lew R, Weaver K, Coldwell C (2015). Partnering with health system operations leadership to develop a controlled implementation trial. Implement Sci.

[17] Bauer MS, Miller CJ, Kim B, Lew R, Stolzmann K, Sullivan J (2019). Effectiveness of Implementing a Collaborative Chronic Care Model for Clinician Teams on Patient Outcomes and Health Status in Mental Health: A Randomized Clinical Trial. JAMA Netw Open.

[18] Miller CJ, Grogan-Kaylor A, Perron BE, Kilbourne AM, Woltmann E, Bauer MS (2013). Collaborative chronic care models for mental health conditions: cumulative meta-analysis and metaregression to guide future research and implementation. Med Care.

[19] Von Korff M, Gruman J, Schaefer J, Curry SJ, Wagner EH (1997). Collaborative management of chronic illness. Ann Intern Med.

[20] Kirchner J (2019). Behavioral health QUERI implementation facilitation training hub VA Quality Enhancement Research Initiative.

[21] Kilbourne AM, Neumann MS, Pincus HA, Bauer MS, Stall RJ (2007). Implementing evidence-based interventions in health care: application of the replicating effective programs framework. Implement Sci.

[22] NVivo 10 (2012). QSR International.

[23] Hsieh HF, Shannon SE (2005). Three approaches to qualitative content analysis. Qual Health Res.

[24] O’Brien BC, Harris IB, Beckman TJ, Reed DA, Cook DA (2014). Standards for reporting qualitative research: a synthesis of recommendations. Acad Med.

[25] Ritchie M, Dollar K, Miller C, Oliver K, Smith J, Lindsay J (2017). Using implementation facilitation to improve Care in the Veterans Health Administration (version 2).

[26] Braun V, Clarke V, Terry G (2012). Thematic analysis. APA handbook of research methods in psychology.

