# Supplementary material for: External facilitators’ perceptions of internal facilitation skills during implementation of collaborative care for mental health teams: a qualitative analysis informed by the i-PARIHS framework
Source: BMC Health Serv Res. 2020 Mar 4;20:165. doi: 10.1186/s12913-020-5011-3 (PMC7057643; doi:10.1186/s12913-020-5011-3)
Supplement: Supplementary file 1 — Additional file 1. Interview Guide. [file 12913_2020_5011_MOESM1_ESM.docx]

**Interview Guide**

“The goal of this interview is to gain your perspective on factors influencing the effectiveness of the internal facilitators that you worked with at your three sites.”

- Ask: are there any questions? When questions have been addressed, ask:
  - Do you consent to be interviewed?
  - Do you consent to be recorded?
  - If yes to both, turn on the tape recorder and state: “thanks, I am now turning the tape recorder on…”
- Following turning on the recorder, state: “Okay, now that we are recording, I need to ask you for the record:
  - Do you consent to be interviewed?
  - Do you consent to be recorded?
  - And please let me know at any point if you would like to turn off the recorder… “

1. What characteristics of your IFs seemed most important to their success and effectiveness as a facilitator?
   1. PROBES:
      1. Project management/improvement skills
         1. E.g., consistency adhering to new processes, admin/technical skills and experience, bandwidth to focus on BHIP, role clarity, ability to delegate responsibilities, prior project management experience?
      2. Team and process skills
         1. E.g., level of respect from team, accessibility (answering emails, attending mtgs), ability to facilitate discussion, invite questions/be transparent, feed info back to team, provide team members w/ autonomy, leadership abilities, interpersonal/communication skills, support/encouragement, supervisory authority over team members, degree of prior establishment within team, prior leadership experience?
      3. Influencing and negotiating skills
         1. E.g., ability to gain stakeholder support, advocate for team/get necessary resources, ability to provide organizational insight/perspective, understand context, distinguish between team-level vs. org-level goals, prior experience influencing/negotiating?
      4. Personal characteristics
         1. E.g., level of commitment, enthusiasm, motivation, patience, self-starter, creativity, flexibility, openness to feedback/critique
2. Did these characteristics vary across the IFs you worked with?

IF YES: How so?

PROBE FOR EACH OF THE FOUR CATEGORIES ABOVE IF NOT MENTIONED.

1. Were there any additional characteristics we haven’t yet discussed that you felt were important to IF success?

IF YES: What were they?

1. What characteristics of your IFs seemed to hinder the facilitation process?
   1. PROBES:
      1. Project management/improvement skills
         1. E.g., consistency adhering to new processes, admin/technical skills and experience, bandwidth to focus on BHIP, role clarity, ability to delegate responsibilities, prior project management experience?
      2. Team and process skills
         1. E.g., level of respect from team, accessibility (answering emails, attending mtgs), ability to facilitate discussion, invite questions/be transparent, feed info back to team, provide team members w/ autonomy, leadership abilities, interpersonal/communication skills, support/encouragement, supervisory authority over team members, degree of prior establishment within team, prior leadership experience?
      3. Influencing and negotiating skills
         1. E.g., ability to gain stakeholder support, advocate for team/get necessary resources, ability to provide organizational insight/perspective, understand context, distinguish between team-level vs. org-level goals, prior experience influencing/negotiating?
      4. Personal characteristics
         1. E.g., level of commitment, enthusiasm, motivation, patience, self-starter, creativity, flexibility, openness to feedback/critique
2. Did these characteristics vary across the IFs you worked with?

IF YES: How so?

PROBE FOR EACH OF THE FOUR CATEGORIES ABOVE IF NOT MENTIONED.

1. Were there any additional IF characteristics you haven’t yet mentioned that may have hindered facilitation efforts?

IF YES: Which ones?

**Wrap-Up**

1. Is there anything we haven’t yet covered that feels important to discuss regarding factors influencing IF effectiveness?
